# Design based synthetic imputation methods for domain mean

**DOI:** 10.1038/s41598-024-53909-0

**Published:** 2024-02-21

**Authors:** Shashi Bhushan, Anoop Kumar, Rohini Pokhrel, M. E. Bakr, Getachew Tekle Mekiso

**Affiliations:** 1 Department of Statistics, University of Lucknow, Lucknow, 226007 India; 2 Department of Statistics, Faculty of Basic Science, Central University of Haryana, Mahendergarh, 123031 India; 3 Department of Mathematics and Statistics, Dr. Shakuntala Misra National Rehabilitation University, Lucknow, India; 4 Department of Statistics and Operations Research, College of Science, King Saud University, P.O. Box 2455, Riyadh, 11451 Saudi Arabia; 5 Department of Statistics, Wachemo University, Hosaina, Ethiopia

**Keywords:** Small area estimation, Missing value, Imputation, Efficiency, Mathematics and computing, Engineering

## Abstract

In real life, situations may arise when the available data are insufficient to provide accurate estimates for the domain, the small area estimation (SAE) technique has been used to get accurate estimates for the variable under study. The problem of missing data is a serious problem that has an impact on sample surveys, but small area estimates are especially prone to it. This paper is a basic effort that suggests design based synthetic imputation methods for the domain mean estimation using simple random sampling in order to address the issue of missing data under SAE. The expression of the mean square error for the proposed imputation methods are obtained up to first order approximation. The efficiency conditions are determined and a thorough simulation study is carried out using artificially generated data sets. An application is included with real data that further supports this study.

## Introduction

The majority of surveys are only intended to offer estimates at the national and/or state/territory geographic levels that are statistically valid and design-based. Implementing and carrying out sample surveys that would produce accurate estimates at levels smaller than state/territory would be extremely difficult and expensive, both in terms of the larger sample sizes needed and the increased burden on survey respondents. Small area estimates are produced using small area estimation (SAE) techniques to get beyond the issue of small sample numbers and outperform the accuracy of direct survey estimates derived from the sample in each small region. Direct, synthetic, and other indirect estimations are some of the techniques used for SAE. The direct estimators solely employ information from the specified region under study. Mostly, they are unbiased, but very unstable having large variation. Indirect and composite estimators are more accurate because they additionally include information from related variables or nearby areas.

The direct estimators have been shown to produce unacceptable large standard errors as a result of asymmetric small samples from the relevant small area. In reality, there may be circumstances when no sample units can be selected from a portion of small domains. Finding indirect (synthetic) estimators, that dramatically increase sample size and subsequently reduce the standard error of the estimator is therefore necessary to achieve appropriate statistical accuracy. According to Gonzalez^1^ “an estimator is called a synthetic estimator if a reliable direct estimator for a large area, covering several small domains, is used to derive an indirect estimate for a small domain, under the assumption that the small areas have the same characteristics as the large area”. Developing indirect estimators for small areas is necessary since there is a lack of sufficient sample data in small geographic areas. Numerous researchers, particularly in the fields of health, agriculture, and poverty, have developed synthetic estimators. According to recent research by Tikkiwal and Ghiya^2^, Pandey and Tikkiwal^3^, Tikkiwal et al.^4^, Ashutosh et al.^5,6^, Bhushan et al.^7^, small area estimators based on auxiliary information outperform those that exclude it.

The issue of missing data is persistent in sample surveys and necessitates quick action to prevent the validity of any conclusions drawn from such data. The properties such as unbiasedness and efficiency of the estimators might both be compromised by the missing data. Imputation of missing data is the preferred and most often used method for dealing with missing data. Rubin^8^ proposed three fundamental conceptions in his landmark work: missing at random (MAR), observed at random (OAR), and parameter distribution (PD). A discrimination between missing at random (MAR) and missing completely at random (MCAR) was provided by Heitjan and Basu^9^. Many renowned writers have addressed the issue of missing data, and different imputation approaches have been used to fill in the gaps. The accessibility of adequate supplementary information is critical for the creation of effective imputations schemes. Numerous prominent researchers, including Rueda et al.^10^, Toutenburg and Srivastava^11^, Toutenburg et al.^12^, Singh and Horn^13^, Prasad^14^, Singh and Deo^15^, Singh^16^, Ahmed et al.^17^, Bhushan and Pandey^18,19^, Bhushan et al.^20,21^, Prasad^22^, Prasad and Yadav^23^, Bhushan and Kumar^24^ have studied in this field and developed imputations and the corresponding estimators for missing data utilizing auxiliary information. In this study, we use the MCAR approach to impute missing data altogether.

Further, in literature, no imputation method is available to solve the issue of missing data under SAE. Therefore, the objectives of this article are:

(i) to propose some fundamental imputations, namely, mean, ratio, logarithmic type for estimating the domain mean;
(ii) to propose Searls type logarithmic imputation methods estimating the domain mean;
(iii) to compare the fundamental imputations with our Searls type logarithmic imputation methods.

Note that while imputing the missing observations, we do not modify the original responses. The methodology and notations used in this study are discussed below.

### Methodology and notations

Consider a specified population $\Phi =\{1, 2,\ldots , N\}$ of the size *N* from which a simple random sample *s* of the size *n* is drawn without replacement. In order to estimate the mean of domain *d*, we use the information collected in the sample. Further, let $r_d$ and *r* be the amount of units responding from chosen $n_d$ and *n* units and let $R_d$ and *R* be the set of units responding in the domain *d* and total population, respectively. Also, ${\bar{R}}_d$ and ${\bar{R}}$ symbolize the set of units non-responding in the domain *d* and total population, respectively. For all units, $i\in R$, the quantity $y_i$ is obtained, but for the units $i \in {\bar{R}}$, the quantities are missing and imputed data must be obtained to finalize the formation of sample data set. Suppose, the imputation is accomplished comprising the additional auxiliary information, *X*, so $X_i$, the value of *X* for unit *i*, is available and positive for all $i \in s$ such that the data $\mathbf {X_s}=\left\{ X_i;~i \in s\right\}$ are available.

To derive the mean square error (MSE) of the consequent synthetic estimators of the proposed synthetic imputation methods, we take the following notations: ${\bar{y}}_{r}={\bar{Y}}(1+\varepsilon _0)$, ${\bar{x}}_{r}={\bar{X}}(1+\varepsilon _1)$, and ${\bar{x}}_{n}={\bar{X}}(1+\varepsilon _2)$, the $\varepsilon 's$ are error terms such that $E(\varepsilon _k)=0,~k=0,1,2$ and $E(\varepsilon _0^2)=f_{r}C_{y}^2$, $E(\varepsilon _1^2)=f_{r}C_{x}^2$, $E(\varepsilon _2^2)=f_{n}C_{x}^2$, $E(\varepsilon _0\varepsilon _1)=f_{r}\rho _{yx}C_{y}C_{x}$, $E(\varepsilon _0\varepsilon _2)=f_{n}\rho _{yx}C_{y}C_{x}$, $E(\varepsilon _1\varepsilon _2)=f_{n}C_{x}^2$, where, $f_{r}=\left( \frac{1}{r}-\frac{1}{N}\right)$ and $f_{n}=\left( \frac{1}{n}-\frac{1}{N}\right)$, $C_{y}$ and $C_{x}$ are the coefficient of variation of study and auxiliary variables, respectively, $\rho _{yx}$ is the correlation coefficient between study and auxiliary variables.

The content that follows is broken up into a few sections. In “Adapted imputation methods” and “Proposed synthetic Searls type logarithmic imputation methods”, respectively, the adapted and proposed imputation methods are presented together with formulae for the mean square error (MSE). In “Efficiency conditions”, a comparison of the various imputation strategies is given. In “Simulation study”, a comprehensive simulation analysis using a few artificial populations is provided, and the main simulation results are explored. In “Real data application”, an actual data application is also provided. In “Conclusions”, this article is concluded with some concluding remarks.

## Adapted imputation methods

Since literature contains no imputation methods to deal with the problem of estimation of mean of domain *d* in the presence of missing data. Therefore, we adapt some conventional imputation methods for the estimation of domain mean.

### Conventional mean imputation method

When information on the auxiliary variables is not available, then the conventional mean imputation method is the obvious choice. When the *i*th sample unit in domain *d* is missing and requires imputation, we suggest the mean imputation of domain mean by amplifying the notations of Lee et al.^25^ for unit value imputation. The synthetic mean imputation technique for domain mean is given by

$$\begin{aligned} y_{{.i_m}}= {\left\{ \begin{array}{ll} y_i&{}\text {if }i \in R\\ {\bar{y}}_{r} &{}\text {if }i \in {\bar{R}} \end{array}\right. } \end{aligned}$$

The consequent synthetic estimator is

$$\begin{aligned} t_{m}&={\bar{y}}_{r} \end{aligned}$$

The MSE of the consequent synthetic mean estimator is

1 $$\begin{aligned} MSE(t_m)&=({\bar{Y}}-{\bar{Y}}_d)^2+{\bar{Y}}^2f_{r}C_{y}^2 \end{aligned}$$

The imputation approaches are distinguished into two schemes when additional auxiliary information is taken into account.

Scheme I: When ${\bar{X}}_d$ is known and ${\bar{x}}_{n,d}$ is used.

Scheme II: When ${\bar{X}}_d$ is known and ${\bar{x}}_{r,d}$ is used.

### Synthetic ratio imputation methods

The ratio imputation method provides efficient results when the study and auxiliary variables are positively correlated. The classical synthetic ratio imputation methods under schemes I and II are defined as

Scheme I

$$\begin{aligned} y_{{.i}_{r_1}}&= {\left\{ \begin{array}{ll} y_i&{}\text {if }i \in R\\ \frac{1}{n-r}\left[ n{\bar{y}}_{r}\left( \frac{{\bar{X}}_d}{{\bar{x}}_{n}}\right) -r{\bar{y}}_{r}\right] &{}\text {if }i \in {\bar{R}} \end{array}\right. } \end{aligned}$$

Scheme II

$$\begin{aligned} y_{{.i}_{r_2}}&= {\left\{ \begin{array}{ll} y_i&{}\text {if }i \in R\\ \frac{1}{n-r}\left[ n{\bar{y}}_{r}\left( \frac{{\bar{X}}_d}{{\bar{x}}_{r}}\right) -r{\bar{y}}_{r}\right] &{}\text {if }i \in {\bar{R}} \end{array}\right. } \end{aligned}$$

The consequent synthetic ratio estimators under above schemes are

$$\begin{aligned} t_{r_1}&={\bar{y}}_{r}\left( \frac{{\bar{X}}_d}{{\bar{x}}_{n}}\right) \\ t_{r_2}&={\bar{y}}_{r}\left( \frac{{\bar{X}}_d}{{\bar{x}}_{r}}\right) \end{aligned}$$

#### Theorem 2.1

*The MSE of the consequent synthetic ratio estimators* $t_{r_j},~j=1,2$ *of the synthetic ratio imputation methods* $y_{{.i}_{r_j}}$ *under schemes I and II is given by*

2 $$\begin{aligned} MSE(t_{r_1})&={\bar{Y}}_d^2\left( f_{r}C_{y}^2+f_{n}C_{x}^2-2f_{n}\rho _{yx}C_{y}C_{x}\right) \end{aligned}$$

3 $$\begin{aligned} MSE(t_{r_2})&={\bar{Y}}_d^2f_{r}\left( C_{y}^2+C_{x}^2-2\rho _{yx}C_{y}C_{x}\right) \end{aligned}$$

### Synthetic logarithmic imputation methods

The proposed synthetic logarithmic imputation methods under schemes I and II are given below.

Scheme I

$$\begin{aligned} y_{{.i}_{l_1}}&= {\left\{ \begin{array}{ll} y_i&{}\text {if }i \in R\\ \frac{1}{n-r}\left[ n{\bar{y}}_{r}\left\{ 1+\theta _{1}\log \left( \frac{{\bar{x}}_{n}}{{\bar{X}}_d}\right) \right\} -r{\bar{y}}_{r}\right] &{}\text {if }i \in {\bar{R}} \end{array}\right. } \end{aligned}$$

Scheme II

$$\begin{aligned} y_{{.i}_{l_2}}&= {\left\{ \begin{array}{ll} y_i&{}\text {if }i \in R\\ \frac{1}{n-r}\left[ n{\bar{y}}_{r}\left\{ 1+\theta _{2}\log \left( \frac{{\bar{x}}_{r}}{{\bar{X}}_d}\right) \right\} -r{\bar{y}}_{r}\right] &{}\text {if }i \in {\bar{R}} \end{array}\right. } \end{aligned}$$

The resulting estimators are calculated under the schemes described above as

$$\begin{aligned} t_{l_1}&={\bar{y}}_{r}\left\{ 1+\theta _1\log \bigg (\frac{{\bar{x}}_{n}}{{\bar{X}}_d}\bigg )\right\} \\ t_{l_2}&={\bar{y}}_{r}\left\{ 1+\theta _2\log \bigg (\frac{{\bar{x}}_{r}}{{\bar{X}}_d}\bigg )\right\} \end{aligned}$$

where $\theta _j$; $j=1,2$ are the suitably chosen scalars.

#### Theorem 2.2

*The* *MSE* *and minimum* *MSE* *of the consequent synthetic estimators* $t_{l_j},~j=1,2$ *of the proposed synthetic imputation methods* $y_{{.i}_{l_j}}$ *under schemes I and II are given by*

$$\begin{aligned} MSE(t_{l_1})&={\bar{Y}}_d^2(f_{r}C_{y}^2+\theta _{1}^2f_{n}C_{x}^2-2\theta _1f_{n}\rho _{yx}C_{y}C_{x})\\ MSE(t_{l_2})&={\bar{Y}}_d^2f_{r}(C_{y}^2+\theta _{2}^2C_{x}^2-2\theta _2\rho _{yx}C_{y}C_{x})\\ minMSE(t_{l_1})&={\bar{Y}}^2_dC_{y}^2\left( f_{r}-f_{n}\rho _{yx}^2\right) \\ minMSE(t_{l_2})&={\bar{Y}}^2_dC_{y}^2f_{r}\left( 1-\rho _{yx}^2\right) \end{aligned}$$

## Proposed synthetic Searls type logarithmic imputation methods

In order to increase the effectiveness of the estimators, Searls^26^ developed a transformation that required multiplying a tuning parameter in the estimators. Therefore, in order to improve the above works, we used a tuning parameter $\delta _j,~j=1,2$ in the synthetic logarithmic imputation methods $y_{{.i}_{l_j}}$ and propose synthetic Searls type logarithmic imputation methods for the mean of domain *d* utilizing auxiliary information in SRS.

The proposed synthetic Searls type logarithmic imputation methods under schemes I and II are given below.

Scheme I

$$\begin{aligned} y_{{.i}_{s_1}}&= {\left\{ \begin{array}{ll} y_i&{}\text {if }i \in R\\ \frac{1}{n-r}\left[ n\delta _1{\bar{y}}_{r}\left\{ 1+\theta _{1}\log \left( \frac{{\bar{x}}_{n}}{{\bar{X}}_d}\right) \right\} -r{\bar{y}}_{r}\right] &{}\text {if }i \in {\bar{R}} \end{array}\right. } \end{aligned}$$

Scheme II

$$\begin{aligned} y_{{.i}_{s_2}}&= {\left\{ \begin{array}{ll} y_i&{}\text {if }i \in R\\ \frac{1}{n-r}\left[ n\delta _2{\bar{y}}_{r}\left\{ 1+\theta _{2}\log \left( \frac{{\bar{x}}_{r}}{{\bar{X}}_d}\right) \right\} -r{\bar{y}}_{r}\right] &{}\text {if }i \in {\bar{R}} \end{array}\right. } \end{aligned}$$

where $\delta _j$, $j=1,2$ are the suitably chosen scalars. The resulting synthetic estimators are calculated under the schemes described above as

$$\begin{aligned} t_{s_1}&=\delta _1{\bar{y}}_{r}\left\{ 1+\theta _1\log \bigg (\frac{{\bar{x}}_{n}}{{\bar{X}}_d}\bigg )\right\} \\ t_{s_2}&=\delta _2{\bar{y}}_{r}\left\{ 1+\theta _2\log \bigg (\frac{{\bar{x}}_{r}}{{\bar{X}}_d}\bigg )\right\} \end{aligned}$$

### Special case

When $\delta _j=1,~j=1,2$, then under schemes I and II, the proposed synthetic Searls type logarithmic imputation methods $y_{{.i}_{s_j}}$ and the corresponding resultant synthetic Searls type logarithmic estimators $t_{s_j}$ deform into the synthetic logarithmic imputation methods $y_{{.i}_{l_j}}$ and the corresponding resultant synthetic logarithmic estimators $t_{l_j}$, respectively.

#### Theorem 3.1

*The* *MSE* *and minimum* *MSE* *of the consequent synthetic estimators* $t_{s_j},~j=1,2$ *of the proposed synthetic imputation methods* $y_{{.i}_{s_j}}$ *under schemes I and II are given by*

$$\begin{aligned} MSE(t_{s_1})&=\left[ \begin{array}{l}{\bar{Y}}_{d}^2+\delta _1^2\left\{ {\bar{Y}}^2_d+f_r{\bar{Y}} _d^2C_y^2+f_n\theta _1^2{\bar{Y}}^2C_x^2+4\theta _1{\bar{Y}}{\bar{Y}}_df_n \rho _{xy}C_xC_y-\theta _1{\bar{Y}}{\bar{Y}}_df_nC_x^2\right\} \\ -2\delta _1\left\{ {\bar{Y}}^2 +\theta _1{\bar{Y}}{\bar{Y}}_df_n\left( \rho _{xy}C_xC_y-\frac{C_x^2}{2}\right) \right\} \end{array}\right] \\ MSE(t_{s_2})&=\left[ \begin{array}{l}{\bar{Y}}_{d}^2+\delta _2^2\left\{ {\bar{Y}}^2_d +f_r{\bar{Y}}_d^2C_y^2+f_r\theta _2^2{\bar{Y}}^2C_x^2+4\theta _2{\bar{Y}}{\bar{Y}}_df_ r\rho _{xy}C_xC_y-\theta _2{\bar{Y}}{\bar{Y}}_df_rC_x^2\right\} \\ -2\delta _2\left\{ {\bar{Y}}^2 +\theta _2{\bar{Y}}{\bar{Y}}_df_r\left( \rho _{xy}C_xC_y- \frac{C_x^2}{2}\right) \right\} \end{array}\right] \\ minMSE(t_{s_1})&={\bar{Y}}^2_d- \frac{Q_1^2}{P_1}\\ minMSE(t_{s_2})&={\bar{Y}}^2_d- \frac{Q_2^2}{P_2} \end{aligned}$$

*where*

$$\begin{aligned} P_1&={\bar{Y}}^2_d+f_r{\bar{Y}}_d^2C_y^2+f_n\theta _1^2{\bar{Y}}^2C_x^2+4\theta _1{\bar{Y}}{\bar{Y}}_df_n \rho _{xy}C_xC_y-\theta _1{\bar{Y}}{\bar{Y}}_df_nC_x^2,\\ Q_1&={\bar{Y}}^2+\theta _1{\bar{Y}}{\bar{Y}}_df_n\left( \rho _{xy}C_xC_y-\frac{C_x^2}{2}\right) ,\\ P_2&={\bar{Y}}^2_d+f_r{\bar{Y}}_d^2C_y^2+f_r\theta _2^2{\bar{Y}}^2C_x^2+4\theta _2{\bar{Y}}{\bar{Y}}_df_r \rho _{xy}C_xC_y-\theta _2{\bar{Y}}{\bar{Y}}_df_rC_x^2,\\ ~~\text {and}~~ Q_2&={\bar{Y}}^2+\theta _2{\bar{Y}}{\bar{Y}}_df_r\left( \rho _{xy}C_xC_y-\frac{C_x^2}{2}\right) . \end{aligned}$$

#### Proof

Consider the proposed consequent synthetic estimator $t_{s_1}$ as

$$\begin{aligned} t_{s_1}&=\delta _1{\bar{y}}_{r}\left\{ 1+\theta _1\log \bigg (\frac{{\bar{x}}_{n}}{{\bar{X}}_d}\bigg )\right\} \end{aligned}$$

We can express the above estimator using the notations established in the previous section as

$$\begin{aligned} t_{s_1}&=\delta _1{\bar{Y}}(1+\varepsilon _0)\left[ 1+{\theta _1}\log \bigg \{\frac{{\bar{X}} (1+\varepsilon _2)}{{\bar{X}}_d}\bigg \}\right] \\&=\delta _1{\bar{Y}}(1+\varepsilon _0)\left[ 1+{\theta _1}\bigg \{\log \left( \frac{{\bar{X}}}{{\bar{X}}_d} \right) +\log (1+\varepsilon _2)\bigg \}\right] \\&=\delta _1{\bar{Y}}(1+\varepsilon _0)\left[ 1+{\theta _1}\bigg \{A+ \left( \varepsilon _2-\frac{\varepsilon _2^2}{2}+\cdots \right) \bigg \}\right] \end{aligned}$$

Simplifying the above expression and neglecting the higher order error terms, we get

$$\begin{aligned} t_{s_1}&=\delta _1{\bar{Y}}\left\{ 1+\varepsilon _0+\theta _1A+\theta _1\left( \varepsilon _2-\frac{\varepsilon _2^2}{2} \right) +\theta _1(A\varepsilon _0+\varepsilon _0\varepsilon _2)\right\} \end{aligned}$$

Subtracting ${\bar{Y}}_d$ on both sides to the above expression, we get

4 $$\begin{aligned} t_{s_3}-{\bar{Y}}_{d}&=\delta _1{\bar{Y}}(1+\theta _1A)-{\bar{Y}}_{d}+\delta _1{\bar{Y}} \left\{ \varepsilon _0+\theta _1\left( \varepsilon _2-\frac{\varepsilon _2^2}{2}\right) +\theta _1(A\varepsilon _0+\varepsilon _0\varepsilon _2)\right\} \end{aligned}$$

Squaring and taking expectation both sides to (4), we get MSE of the estimator $t_{s_1}$ to the first order approximation as

5 $$\begin{aligned} MSE(t_{s_1})&=\left[ \begin{array}{l}\{\delta _1{\bar{Y}}(1+\theta _1A)-{\bar{Y}}_{d}\}^2 +2\delta _1\theta _1{\bar{Y}}\{\delta _1{\bar{Y}}(1+\theta _1A)-{\bar{Y}}_{d}\}f_n \left( \rho _{xy}C_xC_y-\frac{C_x^2}{2}\right) \\ +\alpha ^2{\bar{Y}}^2\left\{ (1+\theta _1A)^ 2f_rC_y^2+\theta _1^2f_nC_x^2+2\theta _1(1+\theta _1A)f_n\rho _{xy}C_xC_y\right\} \end{array}\right] \end{aligned}$$

Under the assumption of Searls logarithmic synthetic estimation ${\bar{Y}}(1+\theta _1A)={\bar{Y}}_d$, the $MSE(t_{s_1})$ can be expressed as

6 $$\begin{aligned} MSE(t_{s_1})&=\left[ \begin{array}{l}{\bar{Y}}_{d}^2+\delta _1^2\left\{ {\bar{Y}}^2_d+f_r{\bar{Y}}_d^2C_y^2 +f_n\theta _1^2{\bar{Y}}^2C_x^2+4\theta _1{\bar{Y}}{\bar{Y}}_df_n\rho _{xy}C_xC_y -\theta _1{\bar{Y}}{\bar{Y}}_df_nC_x^2\right\} \\ -2\delta _1\left\{ {\bar{Y}}^2 +\theta _1{\bar{Y}}{\bar{Y}}_df_n\left( \rho _{xy}C_xC_y-\frac{C_x^2}{2}\right) \right\} \end{array}\right] \nonumber \\&={\bar{Y}}_d^2+\delta _1^2P_1-2\delta _1Q_1 \end{aligned}$$

where

$$\begin{aligned} P_1&={\bar{Y}}^2_d+f_r{\bar{Y}}_d^2C_y^2+f_n\theta _1^2{\bar{Y}}^2C_x^2 +4\theta _1{\bar{Y}}{\bar{Y}}_df_n\rho _{xy}C_xC_y-\theta _1{\bar{Y}}{\bar{Y}}_df_nC_x^2\\ \text {and}~~ Q_1&={\bar{Y}}^2+\theta _1{\bar{Y}}{\bar{Y}}_df_n\left( \rho _{xy}C_xC_y-\frac{C_x^2}{2}\right) . \end{aligned}$$

Partially differentiating (6) regarding $\delta _1$ and equating to zero, we get the optimum value of $\delta _1$ as

$$\begin{aligned} \delta _{1(opt)}=\frac{Q_1}{P_1} \end{aligned}$$

Putting the optimum value of $\delta _1$ from the above expression to (6), we get minimum MSE of the estimator $t_{s_1}$ as

7 $$\begin{aligned} min.MSE(t_{s_1})&={\bar{Y}}_{d}^2-\frac{Q_1^2}{P_1} \end{aligned}$$

Similarly, the first order approximated expressions of MSE and minimum MSE of the proposed synthetic estimator $t_{s_2}$ can be obtained. $\square$

## Efficiency conditions

In the present section, we compare the minimum *MSE* of the proposed synthetic imputation methods with the corresponding minimum *MSE* of the existing synthetic imputation methods under schemes I and II.

### Lemma 4.1

*The proposed synthetic Searls type logarithmic imputation methods* $y_{.i_{s_j}},~j=1,2$ *dominate the synthetic mean imputation method* $y_{.i_{m}}$, *if*

$$\begin{aligned} MSE(t_{s_j})&<MSE(t_m)\implies \frac{Q_j^2}{P_j}>1-\frac{({\bar{Y}}-{\bar{Y}}_d)^2}{{\bar{Y}}_d^2}-\frac{{\bar{Y}}^2}{{\bar{Y}}_d^2}f_{r}C_{y}^2 \end{aligned}$$

### Lemma 4.2

*The proposed synthetic Searls type logarithmic imputation methods* $y_{.i_{s_j}},~j=1,2$ *dominate the synthetic ratio imputation methods* $y_{.i_{r_j}}$ *under schemes I and II, if*

$$\begin{aligned} MSE(t_{s_j})&<MSE(t_{r_j})\implies \frac{Q_j^2}{P_j}>1-f_{r}C_{y}^2-f_{n}C_{x}^2+2f_{n}\rho _{yx}C_{y}C_{x} \end{aligned}$$

### Lemma 4.3

*The proposed synthetic Searls type logarithmic imputation methods* $y_{.i_{s_j}},~j=1,2$ *dominate the synthetic logarithmic imputation methods* $y_{.i_{l_j}}$ *under schemes I and II, if*

$$\begin{aligned} MSE(t_{s_j})&<MSE(t_{l_j})\implies \frac{Q_j^2}{P_j}>1-C_{y}^2(f_{r}-f_{n}\rho _{yx}^2) \end{aligned}$$

The proposed synthetic Searls type logarithmic imputation methods repress the synthetic mean per unit imputation method, synthetic ratio imputation methods and synthetic logarithmic imputation methods, if the aforementioned lemmas are satisfied. The next section verifies the above lemmas utilizing a comprehensive simulation study.

## Simulation study

A simulation study is executed to assess the effectiveness of the suggested synthetic imputation methods in comparison to the adapted synthetic imputation methods. In the simulation procedure, certain symmetrical and asymmetrical populations are produced in accordance with the models employed by Singh and Horn^27^. The model used are as follows:

$$\begin{aligned} y&=5.5+\sqrt{(1-\rho _{xy}^2)}~y^*+\rho _{xy}\left( \frac{S_y}{S_x}\right) x^*\\ x&=5.3+x^* \end{aligned}$$

where $x^*$ and $y^*$ are independent variables for the corresponding distributions. Considering the above models, we have generated the below mentioned populations:

1. A Normal population of size *N*=6000 using $x^*\sim N(12,35)$ and $y^*\sim N(13,45)$ with varying correlation coefficients $\rho _{xy}$=0.1, 0.5, 0.9.
2. A Gamma population of size *N*=6000 using $x^*\sim G(0.02,0.006)$ and $y^*\sim G(0.2,0.011)$ with varying correlation coefficients $\rho _{xy}$=0.1, 0.5, 0.9.

The above populations are divided into 6 equal domains of size 1000. We have drawn a random sample of sizes $(n_1,~n_2,~n_3,~n_4,~n_5,~n_6)=(200,~250,~300,~350,~100,~150)$ from the respective domains and chosen the varying response rates $r_1=(170,~180)$, $r_2=(230,~240)$, $r_3=(270,~280)$, $r_4=(330,~340)$, $r_5=(80,~90)$, and $r_6=(130,~140)$ from the respective samples. The imputation strategy is taken and the MSE of the consequent estimators is computed by utilizing 15,000 iterations. The simulation procedure is explained in the undermentioned steps.

(i) Select a sample *s* of size *n* randomly from the population of size *N*.
(ii) Bring out randomly ($n_d$-$r_d$) sample units through sample *s* every time.
(iii) Impute selected units by considering the proposed imputation methods studied for quantified samples.
(iv) Compute the needed statistics.
(v) Iterated the prior steps 15,000 times.

The empirical (simulated) mean square error (EMSE) and the theoretical mean square error (TMSE). The TMSE is calculated using the MSE expressions of the respective estimators obtained in “Adapted imputation methods” and “Proposed synthetic Searls type logarithmic imputation methods”, while the EMSE is calculated utilizing the following formula:

8 $$\begin{aligned} EMSE(t_{*})&=\frac{1}{15,000}\sum _{i=1}^{15,000}(t_{*}-{\bar{Y}}_d)^2 \end{aligned}$$

where $$t_{*}$$=$$t_{m}$$, $$t_{r_j},~j=1,2$$, $$t_{l_j}$$, $$t_{s_j}$$.

The results of the consequent synthetic estimators for normal and gamma populations are reported in Tables 1 and 2, respectively.

### Key results of simulation study

We interpret the key results of simulation study summarized from Tables 1 to 2 in the following points.

1. The outcomes drawn from normal population for the consequent synthetic estimators are reported in Table 1. These outcomes show that:
  - the EMSE and TMSE of the consequent synthetic ratio estimator $t_{r_1}$ under scheme I decreases with the successive increase in the correlation coefficient $\rho _{xy}$ from 0.1 to 0.9. This tendency in the EMSE and TMSE values of $t_{r_1}$ can be also observed from scheme II for the estimator $t_{r_2}$.
  - the EMSE and TMSE of the consequent synthetic logarithmic estimator $t_{l_1}$ under scheme I decreases with the successive increase in the values of correlation coefficient $\rho _{xy}$ from 0.1 to 0.9. This tendency in the EMSE and TMSE values of $t_{l_1}$ can be also observed from scheme II for the consequent synthetic logarithmic estimator $t_{l_2}$.
  - the EMSE and TMSE of the consequent synthetic Searls type logarithmic estimator $t_{s_1}$ under scheme I decreases with the successive increase in the correlation coefficient $\rho _{xy}$ from 0.1 to 0.9. This tendency in the EMSE and TMSE values of $t_{s_1}$ can be also observed from scheme II for the consequent synthetic Searls type logarithmic estimator $t_{s_2}$.
  - the EMSE and TMSE of the consequent synthetic ratio estimators, synthetic logarithmic estimators, and synthetic Searls type logarithmic estimators decreases with the increase in the responding units $r_d$ under schemes I and II in each domain.
  - the EMSE and TMSE of the consequent synthetic ratio estimators, synthetic logarithmic estimators, and synthetic Searls type logarithmic estimators under both schemes in each domain are observed to be very close to each other.
  - the consequent synthetic Searls type logarithmic estimators $t_{s_j},~j=1,2$ perform better than the adapted synthetic mean estimator $t_{m}$, synthetic ratio estimators $t_{r_j}$, and synthetic logarithmic estimators $t_{l_j}$ under schemes I and II.
2. The similar tendency as observed from the results of Table 1 obtained from normal population for synthetic estimators can also be observed from the results of Table 2 obtained from gamma population for synthetic estimators.
3. Finally, from the results of Tables 1 and 2, the performance of the synthetic ratio estimators, synthetic logarithmic estimators, and synthetic Searls type logarithmic estimators is better under scheme II compared to scheme I.

**Table 1:** EMSE and TMSE of synthetic estimators under normal population.

	$\rho _{xy}$	$r_d$	$t_{m}$		Scheme I						Scheme II					
					$t_{r_1}$		$t_{l_1}$		$t_{s_1}$		$t_{r_2}$		$t_{l_2}$		$t_{s_2}$	
Domains			EMSE	TMSE	EMSE	TMSE	EMSE	TMSE	EMSE	TMSE	EMSE	TMSE	EMSE	TMSE	EMSE	TMSE
1	0.1	170	2.74	2.56	1.94	1.85	1.23	1.19	1.22	1.19	2.04	1.95	1.22	1.19	1.21	1.18
		180	2.70	2.48	1.87	1.78	1.16	1.12	1.15	1.12	1.93	1.84	1.16	1.12	1.15	1.11
	0.5	170	2.30	1.93	1.48	1.41	1.05	1.00	1.04	1.00	1.50	1.43	1.00	0.96	0.98	0.96
		180	2.20	1.85	1.40	1.33	0.97	0.93	0.96	0.93	1.41	1.34	0.94	0.91	0.92	0.90
	0.9	170	1.55	1.36	0.49	0.47	0.44	0.40	0.42	0.40	0.36	0.34	0.27	0.26	0.26	0.26
		180	1.47	1.28	0.41	0.39	0.35	0.32	0.33	0.32	0.33	0.32	0.25	0.24	0.24	0.24
2	0.1	230	2.01	1.89	2.32	2.24	1.47	1.44	1.46	1.43	2.44	2.36	1.46	1.44	1.45	1.43
		240	1.95	1.81	2.23	2.15	1.38	1.35	1.37	1.35	2.30	2.22	1.38	1.35	1.37	1.35
	0.5	230	3.20	3.17	1.74	1.67	1.23	1.19	1.22	1.19	1.76	1.69	1.18	1.14	1.17	1.14
		240	3.11	3.09	1.65	1.58	1.14	1.10	1.13	1.10	1.66	1.59	1.11	1.07	1.10	1.07
	0.9	230	4.58	4.54	0.56	0.54	0.49	0.46	0.48	0.46	0.41	0.39	0.31	0.30	0.30	0.30
		240	4.50	4.46	0.47	0.45	0.39	0.38	0.38	0.37	0.38	0.37	0.29	0.28	0.27	0.28
3	0.1	270	2.61	2.55	2.41	2.32	1.54	1.49	1.52	1.48	2.54	2.44	1.52	1.49	1.50	1.48
		280	2.53	2.48	2.30	2.23	1.43	1.40	1.42	1.40	2.37	2.30	1.42	1.40	1.41	1.39
	0.5	270	1.68	1.60	1.63	1.56	1.16	1.11	1.15	1.11	1.65	1.58	1.11	1.07	1.10	1.06
		280	1.63	1.52	1.54	1.48	1.07	1.03	1.06	1.03	1.55	1.49	1.03	1.00	1.02	1.00
	0.9	270	1.60	1.60	0.47	0.45	0.41	0.39	0.40	0.39	0.34	0.33	0.26	0.25	0.25	0.25
		280	1.57	1.52	0.39	0.38	0.33	0.31	0.32	0.31	0.32	0.31	0.24	0.23	0.22	0.23
4	0.1	330	5.00	4.91	1.76	1.70	1.12	1.09	1.11	1.09	1.85	1.79	1.11	1.09	1.10	1.09
		340	4.81	4.83	1.69	1.64	1.05	1.03	1.04	1.02	1.74	1.69	1.04	1.03	1.02	1.02
	0.5	330	4.80	4.80	1.33	1.28	0.95	0.91	0.94	0.91	1.35	1.29	0.90	0.87	0.89	0.87
		340	4.77	4.75	1.25	1.21	0.88	0.84	0.86	0.84	1.27	1.22	0.84	0.82	0.83	0.82
	0.9	330	3.28	3.24	0.44	0.42	0.39	0.36	0.37	0.36	0.32	0.30	0.24	0.23	0.24	0.23
		340	3.19	3.16	0.37	0.35	0.32	0.29	0.30	0.29	0.30	0.29	0.23	0.22	0.22	0.22
5	0.1	80	3.44	3.09	2.54	2.37	1.62	1.52	1.60	1.52	2.68	2.49	1.60	1.52	1.58	1.51
		90	2.89	3.23	2.44	2.28	1.51	1.43	1.50	1.43	2.51	2.35	1.51	1.43	1.50	1.42
	0.5	80	1.75	1.57	1.67	1.56	1.18	1.11	1.17	1.11	1.69	1.58	1.13	1.06	1.12	1.06
		90	1.62	1.62	1.57	1.47	1.09	1.03	1.08	1.02	1.59	1.49	1.06	1.00	1.05	1.00
	0.9	80	2.52	2.10	0.47	0.44	0.42	0.37	0.40	0.37	0.34	0.32	0.26	0.24	0.25	0.24
		90	2.22	2.02	0.39	0.37	0.33	0.30	0.32	0.30	0.32	0.30	0.24	0.23	0.23	0.23
6	0.1	130	3.97	3.81	2.49	2.43	1.58	1.56	1.57	1.55	2.63	2.55	1.58	1.56	1.57	1.55
		140	3.81	3.73	2.39	2.33	1.48	1.46	1.47	1.46	2.47	2.40	1.48	1.46	1.47	1.46
	0.5	130	5.15	5.06	1.64	1.75	1.17	1.24	1.16	1.24	1.67	1.77	1.11	1.19	1.10	1.19
		140	4.76	4.68	1.55	1.65	1.08	1.15	1.07	1.15	1.56	1.67	1.04	1.12	1.03	1.12
	0.9	130	5.01	4.74	0.47	0.54	0.41	0.46	0.40	0.46	0.34	0.39	0.25	0.30	0.24	0.30
		140	4.75	4.66	0.38	0.45	0.32	0.37	0.31	0.37	0.31	0.37	0.24	0.28	0.23	0.28

**Table 2:** EMSE and TMSE of synthetic estimators under gamma population.

Domains	$\rho _{xy}$	$r_d$	$t_{m}$		Scheme I						Scheme II					
					$t_{r_1}$		$t_{l_1}$		$t_{s_1}$		$t_{r_2}$		$t_{l_2}$		$t_{s_2}$	
			EMSE	TMSE	EMSE	TMSE	EMSE	TMSE	EMSE	TMSE	EMSE	TMSE	EMSE	TMSE	EMSE	TMSE
1	0.1	170	1.38	1.31	3.34	3.23	1.18	1.14	1.17	1.14	3.66	3.54	1.17	1.14	1.16	1.14
		180	1.36	1.24	3.29	3.16	1.12	1.08	1.11	1.07	3.46	3.33	1.10	1.07	1.09	1.07
	0.5	170	1.75	1.44	2.09	2.00	0.93	0.89	0.92	0.89	2.23	2.13	0.88	0.85	0.87	0.85
		180	1.64	1.38	2.04	1.94	0.86	0.82	0.85	0.82	2.11	2.01	0.83	0.80	0.82	0.80
	0.9	170	1.72	1.46	0.46	0.43	0.36	0.34	0.35	0.34	0.35	0.33	0.23	0.22	0.22	0.22
		180	1.60	1.40	0.39	0.37	0.30	0.27	0.29	0.27	0.33	0.31	0.22	0.20	0.21	0.20
2	0.1	230	3.01	2.89	2.87	2.78	1.02	0.98	1.01	0.98	3.14	3.04	1.01	0.98	1.00	0.98
		240	2.95	2.83	2.82	2.72	0.96	0.92	0.95	0.92	2.98	2.86	0.95	0.92	0.94	0.92
	0.5	230	4.55	4.17	1.70	1.64	0.76	0.73	0.75	0.73	1.81	1.74	0.72	0.70	0.71	0.70
		240	4.50	4.11	1.66	1.58	0.71	0.67	0.70	0.67	1.72	1.64	0.68	0.66	0.67	0.66
	0.9	230	4.39	4.38	0.34	0.33	0.28	0.26	0.27	0.26	0.26	0.25	0.17	0.16	0.16	0.16
		240	4.35	4.31	0.29	0.28	0.22	0.20	0.21	0.20	0.25	0.24	0.16	0.15	0.15	0.15
3	0.1	270	1.70	1.49	3.36	3.28	1.19	1.16	1.18	1.16	3.69	3.59	1.18	1.16	1.17	1.16
		280	1.64	1.42	3.31	3.21	1.12	1.09	1.11	1.09	3.49	3.38	1.11	1.09	1.10	1.09
	0.5	270	1.68	1.52	2.09	2.01	0.93	0.90	0.92	0.89	2.23	2.14	0.88	0.86	0.87	0.86
		280	1.57	1.45	2.03	1.95	0.86	0.83	0.85	0.83	2.11	2.02	0.83	0.81	0.82	0.81
	0.9	270	1.55	1.34	0.45	0.43	0.36	0.33	0.35	0.33	0.34	0.33	0.22	0.21	0.21	0.21
		280	1.42	1.27	0.38	0.36	0.29	0.27	0.28	0.27	0.32	0.31	0.21	0.20	0.20	0.20
4	0.1	330	2.32	2.04	3.46	3.37	1.22	1.19	1.21	1.19	3.79	3.69	1.21	1.19	1.20	1.19
		340	2.27	1.97	3.39	3.30	1.15	1.12	1.14	1.12	3.57	3.47	1.14	1.12	1.13	1.12
	0.5	330	2.25	2.00	2.15	2.06	0.95	0.92	0.94	0.92	2.29	2.20	0.90	0.88	0.89	0.88
		340	2.12	1.94	2.08	1.99	0.88	0.85	0.87	0.85	2.16	2.07	0.85	0.83	0.84	0.83
	0.9	330	1.80	1.51	0.46	0.44	0.37	0.34	0.36	0.34	0.35	0.33	0.23	0.22	0.22	0.22
		340	1.75	1.45	0.39	0.37	0.30	0.27	0.29	0.27	0.33	0.31	0.22	0.20	0.21	0.20
5	0.1	80	3.60	3.48	2.89	2.73	1.02	0.97	1.01	0.96	3.17	2.99	1.01	0.96	1.00	0.96
		90	3.54	3.42	2.84	2.67	0.96	0.91	0.95	0.91	3.00	2.81	0.95	0.91	0.94	0.91
	0.5	80	3.46	3.23	1.84	1.68	0.82	0.75	0.80	0.75	1.96	1.79	0.76	0.72	0.75	0.72
		90	3.32	3.16	1.79	1.63	0.76	0.69	0.74	0.69	1.86	1.68	0.72	0.68	0.71	0.67
	0.9	80	2.08	1.93	0.44	0.37	0.35	0.28	0.33	0.28	0.33	0.28	0.21	0.18	0.20	0.18
		90	1.95	1.87	0.37	0.31	0.28	0.23	0.27	0.23	0.31	0.26	0.20	0.17	0.18	0.17
6	0.1	130	1.95	1.78	2.86	2.91	1.01	1.03	1.00	1.03	3.13	3.18	1.00	1.03	0.98	1.03
		140	1.89	1.72	2.80	2.84	0.96	0.97	0.94	0.97	2.95	3.00	0.94	0.97	0.92	0.96
	0.5	130	1.70	1.28	1.82	1.84	0.80	0.82	0.79	0.82	1.94	1.96	0.75	0.78	0.73	0.78
		140	1.62	1.21	1.76	1.78	0.75	0.76	0.73	0.76	1.83	1.84	0.71	0.74	0.70	0.74
	0.9	130	1.30	1.11	0.42	0.42	0.34	0.32	0.32	0.32	0.32	0.32	0.21	0.21	0.20	0.21
		140	1.17	1.05	0.36	0.35	0.28	0.26	0.26	0.26	0.30	0.30	0.20	0.19	0.19	0.19

## Real data application

Like most other Indian states, Uttar Pradesh is separated into a several districts for the purpose of taking taxes and conducting other administrative and agricultural works. Each district is further separated into a number of tehsils, and each tehsil is further separated into several blocks. Blocks are referred to as small domains in this study.

Since the area used for cultivation determines the yield of every crop. Therefore, for applications using real data, we take into account the problem of estimating agricultural output for various blocks in the Agra district of Uttar Pradesh. Six blocks in the Agra district are referred as small domains. The amount of Bajra crop produced (in tonnes) for the agricultural season 2021–2022 is regarded as the study variable *y*, whilst the area of Bajra crop produced (in hectares) for the agricultural season 2021–2022 is regarded as the auxiliary variable *x*. Various information regarding the blocks of Agra district are reported in Table 3, whereas for easy reference, the parameters for each domain are shown in Table 4.

**Table 3:** Total production and area under Bajra crop in Blocks of Agra district for agricultural season 2021–2022.

S. No.	Blocks of Agra District	Number of villages in blocks	Total production (in tonne) under the Bajra crop in 2021–2022 ($Y_d$)	Total area (in hectare) under the Bajra crop in 2021–2022 ($X_d$)
1	Akola	38	12,516	5289
2	Broli Aheer	53	18,574	8125
3	Fatehabad	66	30,507	12628
4	Jaitpur Kalan	45	19,990	8165
5	Sainya	44	14914	7016
6	Shamshabad	53	26,111	10,162
	Total	299	122,612	51,385

**Table 4:** Population parameters for different domains.

Domains	$N_d$	${\bar{Y}}_d$	${\bar{X}}_d$	$S_{y_d}$	$S_{x_d}$	$\rho _{yx_d}$
1	38	329.37	139.18	248.07	99.73	0.965
2	53	350.45	153.30	334.28	142.61	0.985
3	66	462.23	191.33	422.58	165.61	0.986
4	45	444.22	181.44	263.40	115.00	0.983
5	44	338.95	159.45	223.79	99.51	0.982
6	53	492.66	191.74	318.35	124.93	0.987

From the domain sizes $(N_1,~N_2,~N_3,~N_4,~N_5,~N_6)=(38,~53,~66,~45,~44,~53)$ mentioned in Table 4, we have selected samples $(n_1,~n_2,~n_3,~n_4,~n_5,~n_6)=(8,~11,~13,~9,~9,~11)$, respectively. Out of these selected samples, the responding units are taken as $r_1=(5,~7)$, $r_2=(7,~9)$, $r_3=(9,~11)$, $r_4=(5,~7)$, $r_5=(5,~7)$, and $r_6=(7,~9)$, respectively. Taking the parameters of domain given in Tables 3 and 4, we have computed the MSE of the proposed synthetic estimators.

The results based on the real data for synthetic estimators are reported in Table 5, respectively, which show the dominance of the proposed synthetic Searls type logarithmic imputation methods over the corresponding synthetic mean, ratio, and logarithmic type imputation methods. Under both schemes, the proposed synthetic imputation methods outperform the corresponding synthetic mean, ratio, and logarithmic type imputation methods. The MSE of the adapted and proposed synthetic estimators decreases as the responding units increase under both schemes in each domain. Moreover, the adapted synthetic ratio imputations, synthetic logarithmic imputations and the proposed synthetic Searls type logarithmic imputations perform better in scheme II compared to scheme I.

**Table 5:** MSE of synthetic estimators for real population.

Domains	$r_d$	$t_{m}$	Scheme I			Scheme II		
			$t_{r_1}$	$t_{l_1}$	$t_{s_1}$	$t_{r_2}$	$t_{l_2}$	$t_{s_2}$
1	5	7033.69	261.82	261.80	261.57	15.60	15.58	14.42
	7	6883.32	162.93	162.92	162.68	13.54	13.52	12.65
2	7	4240.42	296.41	296.39	296.23	17.66	17.63	16.33
	9	4090.06	184.46	184.44	184.17	15.33	15.30	14.32
3	9	4282.75	515.64	515.61	515.42	30.72	30.68	28.40
	11	4132.39	320.89	320.86	320.63	26.67	26.62	24.91
4	5	2587.61	476.25	476.22	476.01	28.38	28.33	26.23
	7	2437.25	296.38	296.35	296.13	24.63	24.59	23.01
5	5	5653.49	277.28	277.26	277.06	16.52	16.50	15.27
	7	5503.13	172.55	172.54	172.27	14.34	14.32	13.40
6	7	8622.10	585.77	585.74	585.53	34.90	34.85	32.26
	9	8471.74	364.53	364.50	364.36	30.29	30.25	28.30

## Conclusions

In the current article, we have adapted synthetic mean, ratio, and logarithmic imputation methods, while proposing synthetic Searls type logarithmic imputation methods for the estimation of domain mean in the case of missing data under simple random sampling. The algebraic expressions of MSE for the proposed imputation methods are derived to first order approximation. The algebraic conditions are obtained by comparing the MSE expressions of the proposed and adapted imputations. Furthermore, a comprehensive simulation is executed using a deliberately drawn normal (symmetric) and gamma (asymmetric) population in order to assess the performance of the suggested imputation approaches. The EMSE and TMSE obtained in simulation study show that for varying amounts of correlation coefficient as well as responding units in each domain, the suggested synthetic Searls type logarithmic imputation techniques excel compared to the adapted synthetic mean, ratio, and logarithmic imputation methods. Further, from the results of Tables 1 and 2, the EMSE and TMSE of the adapted and suggested estimators are observed to be very close to each other under both the schemes in each domain. In addition, an actual data set based on the production of Bajra crops in the Agra district of Uttar Pradesh, India, is also used to demonstrate the applicability of the suggested imputation approaches. The results of the real data also favour the suggested imputations compared to the adapted imputations. Therefore, under SAE, if missing data is identified, survey practitioners may be advised to employ the suggested imputation procedures.

## Data Availability

All data generated or analysed during this study are included in this published article.
